# Effective reduction of *Salmonella* Enteritidis in broiler chickens using the UPWr_S134 phage cocktail

**DOI:** 10.3389/fmicb.2023.1136261

**Published:** 2023-04-27

**Authors:** Marta Kuźmińska-Bajor, Paulina Śliwka, Paweł Korzeniowski, Maciej Kuczkowski, David Sáez Moreno, Anna Woźniak-Biel, Emilia Śliwińska, Krzysztof Grzymajło

**Affiliations:** ^1^Department of Biotechnology and Food Microbiology, Faculty of Biotechnology and Food Sciences, Wrocław University of Environmental and Life Sciences, Wrocław, Poland; ^2^Department of Epizootiology and Clinic of Birds and Exotic Animals, Faculty of Veterinary Medicine, Wrocław University of Environmental and Life Sciences, Wrocław, Poland; ^3^Centre of Biological Engineering, University of Minho, Braga, Portugal; ^4^LABBELS Associate Laboratory, Guimarães, Braga, Portugal; ^5^Department of Biochemistry and Molecular Biology, Faculty of Veterinary Medicine, Wrocław University of Environmental and Life Sciences, Wrocław, Poland

**Keywords:** *Salmonella*, phage cocktail, antibacterial agent, poultry, biocontrol

## Abstract

*Salmonella* is a poultry-associated pathogen that is considered one of the most important zoonotic bacterial agents of contaminated food of animal origin including poultry products. Many efforts are taken to eliminate it from the food chain, and phages are one of the most promising tools to control *Salmonella* in poultry production. We investigated the usefulness of the UPWr_S134 phage cocktail in reducing *Salmonella* in broiler chickens. For this purpose, we analyzed the survivability of phages in the harsh environment encountered in the chicken gastrointestinal tract, which has low pH, high temperatures, and digestive activity. Phages in the cocktail UPWr_S134 showed the ability to remain active after storage at temperatures ranging from 4 to 42°C, reflecting temperatures of storage conditions, broiler handling, and the chicken body, and exhibited robust pH stability. We found that although simulated gastric fluids (SGF) caused phage inactivation, the addition of feed to gastric juice allows maintenance of UPWr_S134 phage cocktail activity. Further, we analyzed UPWr_S134 phage cocktail anti-*Salmonella* activity in live animals such as mice and broilers. In an acute infection model in mice, the application of doses of 10^7^ and 10^14^ PFU/ml UPWr_S134 phage cocktail resulted in delaying symptoms of intrinsic infection in all analyzed treatment schedules. In *Salmonella*-infected chickens orally treated with the UPWr_S134 phage cocktail the number of pathogens in internal organs in comparison to untreated birds was significantly lower. Therefore we concluded that the UPWr_S134 phage cocktail could be an effective tool against this pathogen in the poultry industry.

## Introduction

Poultry production, which has an essential place in terms of food safety and nutrition, is the fastest growing agricultural sub-sector in developing and developed countries. It is expected that factors such as population growth, income level growth, and urbanization will contribute to the growth of poultry production in the future (Canton, 2021). Consumption of *Salmonella*-contaminated animal products is responsible for 3% of bacterial foodborne diseases worldwide, with about 80 million infections and 155,000 deaths (El-Saadony et al., 2022). Non-typhoid *Salmonella enterica* serovars such as *S.* Enteritidis and *S.* Typhimurium are among the most frequently reported foodborne pathogens worldwide [EFSA and ECDC (European Food Safety Authority and European Centre for Disease Prevention and Control), 2021]. Poultry products such as meat and eggs have been identified as among the most important food vehicles for infections caused by these two *Salmonella* serovars (Wessels et al., 2021). Within the poultry flock, *Salmonella* can be transmitted between birds *via* the fecal-oral route which is a consequence of the presence of asymptomatic carriers in the herd and the large scale of production, which increases the likelihood of *Salmonella* occurrence (Nale et al., 2021). In addition to horizontal transmission, during slaughtering and meat processing there is a risk of carcass-to-carcass contamination with *Salmonella* (Smith et al., 2018). Due to the fact that contamination with *Salmonella* can occur at each step of the poultry production process, many efforts are taken to eliminate *Salmonella* from this cycle to prevent and control this pathogen in humans. The prevention of infections caused by *Salmonella* raises costs of poultry production due to the use of biosecurity measures such as vaccination, feed additives and monitoring (Thames and Sukumaran, 2020; Ruvalcaba-Gómez et al., 2022). In the event of confirmed infections with human *Salmonella* serovars, certain official actions are applied such as flock depopulation, egg pasteurizations or other restrictions (EFSA BIOHAZ Panel (EFSA Panel on Biological Hazards) et al., 2019). Despite the fact that the implementation of interventions programs resulted in a decrease in the occurrence of *Salmonella* in poultry flocks, the prevalence of various *Salmonella* serovars among live birds is still high and ranges from 6 to 30% depending on national regulations (Antunes et al., 2003; Liljebjelke et al., 2005; Jordan et al., 2006; van de Giessen et al., 2006; Gutierrez et al., 2009; Fearnley et al., 2011; Hue et al., 2011; Hyeon et al., 2011; Yang et al., 2011; Srinivasan et al., 2014; EFSA and ECDC, 2022). On the other hand, the massive use of antimicrobials in poultry production leads to the emergence of *Salmonella* strains resistant to antimicrobials such as ampicillin, sulfonamides, tetracyclines and (fluoro)quinolones (De Mesquita Souza Saraiva et al., 2022). Multi-drug resistance was observed at a very high level in *Salmonella* spp. recovered from carcasses of broilers and was estimated to be 53.6% (EFSA and ECDC, 2022). Virulence determinants combined with antimicrobial resistance, and biofilm formation capabilities of *Salmonella* have been an emerging public health threat. The spread of such resistant strains and genes through the food chain is a growing area of public health concern (Karabasanavar et al., 2020).

Recently, the worldwide trends are to limit the usage of antibiotics and encourage the use of alternatives to antibiotics to improve poultry production and human health by using safe and natural products (Gomes et al., 2022). Phage therapy is among the most promising approaches that could replace antibiotics. Phages are found basically everywhere and are considered an environmentally friendly, cost-effective, and sustainable antimicrobial approach (Khan and Rahman, 2022). They are considered as safe as they can target bacteria precisely without interaction with human cells or the surrounding microbiota (Uyttebroek et al., 2022). Phages are self-replicating and self-limiting as they multiply only at the site of infection where the host bacteria are located and they are eliminated from the individual gradually when the bacterial infection is cleared (Forde and Hill, 2018). At present, phages are extensively explored, which is reflected in the ever-increasing number of studies on phage usage as therapeutic or preventive agents, and agents in biocontrol, biosanitation and biopreservation (Kazi and Annapure, 2016). Currently, the application of lytic phages for controlling *Salmonella* in poultry is still in its early stage, although it is not new (Borie et al., 2008; Bardina et al., 2012; Clavijo et al., 2019).

The current study aimed to test the usefulness of the UPWr_S134 phage cocktail in reducing *Salmonella* in poultry. For this purpose, we assessed the UPWr_S134 phage cocktail’s pH and thermal tolerance and the ability to overcome the low pH of gastric juice in the presence of feed. We tested the effectiveness of the UPWr_S134 phage cocktail in reducing the symptoms of *S.* Enteritidis 327 lux infection in a murine model and the number of *Salmonella* in experimentally infected broiler chickens. Our results indicate that this phage cocktail is a promising anti-*Salmonella* tool in poultry production and can be used without the addition of an antacid.

## Materials and methods

### Bacterial strains and conditions of culture

For phage propagation, isolated from diseased chickens *Salmonella* Enteritidis (*S.* Enteritidis) strain A28 from the Strain Collection of the Department of Epizootiology and Clinic of Bird and Exotic Animals, Wrocław University of Environmental and Life Sciences was used in this study. For experimental infections, *S.* Enteritidis 327 lux was used. This strain was originally collected from broiler chicken (Kisiela et al., 2006), modified according to Riedel et al. (2007), and described elsewhere (Kuźmińska-Bajor et al., 2015). Briefly, *S.* Enteritidis 327 lux strain was modified with the chromosomal integration vector *p16Slux* containing the *lux* operon from *Photorhabdus luminescens* and the erythromycin resistance gene. *S.* Enteritidis 327 lux exhibits resistance to erythromycin and the capacity for bioluminescence, and these modifications did not affect invasiveness in mice and chickens (Kuźmińska-Bajor et al., 2012, 2015). All *S.* Enteritidis strains used in this study were stored with 20% (*v*/*v*) glycerol at − 80°C. To reactivate them, strains were cultivated in Luria-Bertani (LB) broth (Sigma-Aldrich, Germany), under aerobic conditions at 37°C overnight with shaking at 150 rpm. *S.* Enteritidis 327 lux culture was supplemented with erythromycin (0.2 mg/ml).

### Bacteriophages

The bacteriophages used in this study, UPWr_S1, UPWr_S3 and UPWr_S4, were isolated from samples of sewage. Taxonomically, UPWr_S1, UPWr_S3 and UPWr_S4 belong to the genus *Jerseyvirus* within the *Siphoviridae* family and were described elsewhere (Kuźmińska-Bajor et al., 2021). *In vitro* tests revealed lysis effectiveness rates of 41, 82, and 55% for UPWr_S1, UPWr_S3 and UPWr_S4 phages on 67 *Salmonell*a strains belonging to 6 serovars. *In silico* analysis showed that all gene products of these phages had no relevance to any known virulence, toxin, or pathogen-associated protein family or gene products of *Salmonella* strains or any other pathogens. Due to the characteristics of these phages, including their activity against different serovars, as well as great antibiofilm potential (Korzeniowski et al., 2022), they were considered a promising tool in combating *Salmonella* and hence were chosen to conduct *in vivo* phage efficacy experiments in mice and chickens. The amplification of UPWr_S1, UPWr_S3 and UPWr_S4 phages was performed on the *S.* Enteritidis A28 host strain. Phages were amplified using a method described elsewhere (Kuźmińska-Bajor et al., 2021). Briefly, bacterial cultures were prepared by inoculation of 10 ml of LB broth with a single colony following overnight incubation at 37°C with shaking at 150 rpm. After incubation, 0.5 ml of ml of phage lysate was added and the culture was continued overnight at 37°C. In the next step, the bacterial culture was centrifuged for 10 min at 5000 × *g* to remove any remaining cell debris and then filtered through 0.22 μm pore size syringe filters. The 5 ml of resulting phage lysate, from the first step of propagation, was added to 150 ml of host culture (OD_600nm_ = 0.2) and incubated overnight at 37°C. Then, the centrifugation and filtration steps were repeated. Bacteriophage titer was determined using the routine test dilution method (Adams, 1959). As a phage mixture, cocktail UPWr_S134 containing phages UPWr_S1, UPWr_S3, and UPWr_S4 was formulated by combining in an equal ratio of 1:1:1.

### pH and thermal tolerance of phage cocktail UPWr_S134

The stability of the UPWr_S134 phage cocktail at titer 10^8^ PFU/ml was tested to determine phage survival at temperatures (4–42°C) representing temperatures of those storage conditions, broiler handling and the chicken body and over a wide pH (2–13).

For stability at different temperature conditions, the working stock of the UPWr_S134 phage cocktail was incubated for 2 weeks at 4 different temperatures 4, 20, 36 and 42°C. 4°C corresponds to storage condition, 20°C corresponds to the temperature of broiler handling during at least half of the broiler production cycle, 36°C to slightly higher temperature of post-hatch handling and 42°C to broilers’ deep body temperature, respectively. Phage titer at each temperature was determined on the 7th and 14th days of incubation by using the double-layer agar method (Adams, 1959).

Stability studies for acidic and alkaline conditions were conducted according to the methods described by Lu et al. (2020). Briefly, the phage cocktail was adjusted with 1 M NaOH or HCl (Sigma-Aldrich), to yield a pH range of 2–13, and incubated at 37°C for 1 h to determine phage survival using the double-layer agar method. All experiments were performed in triplicate.

### UPWr_S134 survival in simulated gastric juice conditions (SGF)

Simulated gastric fluid (SGF) was used to test phage sensitivity to intragastric conditions according to a method described previously (Tang et al., 2013). The experiment was performed providing three distinct conditions: (i) control SGF representing natural stomach acidity, (ii) neutralized low pH of SGF by the addition of 14% CaCO_3_ as an antacid, and (iii) SGF containing 30% m/v chicken feed Brojler Grower I (Tasomix, Poland) as a simulation of the chicken stomach contents during the digestion process. 1 ml of the 10^8^ PFU/ml UPWr_134 phage cocktail was added to 9 ml of pre-warmed SGF and incubated at 42°C for 2 h. 100 μl were taken every 15 min to measure the survival rate of phages. To measure the phage titer, samples were tenfold serial diluted in LB medium and spotted on the lawn of *S.* Enteritidis A28. The plates were incubated at 37°C overnight and observed for the formation of plaques. This experiment was carried out in triplicate.

### *In vivo* assay in the mouse model

The potential for reduction of *Salmonella* by phage cocktail UPWr_S134 was examined in BALB/c female mice (Mossakowski Medical Research Centre Polish Academy of Sciences, Warsaw, Poland) experimentally infected with 10^7^ colony-forming units (CFU) of *S.* Enteritidis 327 lux strain. All experimental work applying the mouse model of infection was approved by the Local Ethical Committee for Animal Experimentation (protocol code 115/2015; Wrocław, Poland) and performed according to the legal requirements. In our previous research, it was demonstrated that all mice infected with this particular dose of *S.* Enteritidis 327 lux strain developed a typhoid-like disease course always resulting in death, preceded by luminescent signals recorded within 24 h, in a period of time ranging from 8 to 11 days post-infection (Kuźmińska-Bajor et al., 2015). Based on these findings, mice that developed luminescence and clinical symptoms of *Salmonella* infection including lethargy, ruffled fur, or ataxia were sacrificed. Bacteria and phages were delivered by oral gavage *via* a feeding needle, which allows the deposition of the microorganism directly into the mouse’s stomach. Mice at age 6–8 weeks were divided into 12 experimental groups, as shown in Table 1, and 7 mice were assigned to each group. In this study, four groups not treated with phage cocktail were prepared along with one infected with 10^7^ CFU of *S.* Enteritidis 327 lux strain in 100 μl of phosphate buffer saline (PBS; group 1), one treated with 10^7^ PFU/animal in 100 μl of PBS phage cocktail UPWr_S134 (group 2), one with phage cocktail UPWr_S134 at a dose of 10^14^ PFU in 100 μl of PBS per animal (group 3) and the uninfected and untreated animals (group 12). Eight *Salmonella* groups were infected with the dose of 10^7^ CFU per mouse and treated with the UPWr_S134 phage cocktail. Four of the *Salmonella*-infected groups were treated with the dose of 10^7^ PFU per mouse (groups 4, 6, 8, 10). Group 4 was treated with UPWr_S134 phage cocktail on day 7 post infection (p.i.). Group 6 was gavaged once a day starting on the 7th day p.i. to developed a typhoid-like disease course. Group 8 was firstly phage-gavaged on the day of infection, following the treatment from the 7th day post infection to the day of euthanasia of each mouse. Group 10 was treated with phage cocktail UPWr_S134, each day post infection with the first dose given 1 h after inoculation with *S.* Enteritidis 327 lux. Four groups infected with *Salmonella* were phage-treated with the dose of 10^14^ PFU per mouse (groups 5, 7, 9, 11) similarly differing in terms of the phage therapy scheme. Group 5 was treated with phages on day 7 p.i. Group 7 was gavaged once a day starting on the 7th day p.i. to the death of particular mice. Group 9 was firstly phage-inoculated on the day of infection, following the treatment from the 7th day post infection to the day of euthanasia. Group 11 was treated with the phage cocktail UPWr_S134 daily starting on the day of *S.* Enteritidis 327 lux infection. The plan of the phage cocktail administration is shown in Table 1. On day 21, all surviving animals were weighed and humanely killed by cervical dislocation, and all test animals were subjected to gross necropsy and dissected.

**Table 1:** Groups of mice receiving UPWr_S134 phage cocktail after being challenged with a lethal dose of *Salmonella* Enteritidis 327 lux.

Group^a^	Doses		Treatment schedule of bacteriophages [d.p.i.]^n^
	UPWr_S134 [PFU]	*Salmonella* Enteritidis 327 lux [CFU]	
1^b^	–	1*10^7^	–
2^c^	1*10^7^	–	0
3^d^	1*10^14^	–	0
4^e^	1*10^7^	1*10^7^	7
5^f^	1*10^14^	1*10^7^	7
6^g^	1*10^7^	1*10^7^	7, 8, 9
7^h^	1*10^14^	1*10^7^	7, 8, 9
8^i^	1*10^7^	1*10^7^	0, 7, 8, 9, 10, 11
9^j^	1*10^14^	1*10^7^	0, 7, 8, 9, 10, 11, 12
10^k^	1*10^7^	1*10^7^	daily
11^l^	1*10^14^	1*10^7^	daily
12^m^	–	–	–

a Each group consisted of 7 mice.

b Control positive, infected with *S.* Enteritidis 327 lux.

c Control negative, 1*10^7^ CFU/bacteriophage treated mouse.

d Control negative, 1*10^14^ CFU/bacteriophage treated mouse.

e Group infected with *S.* Enteritidis 327 lux and bacteriophage treated with a single dose of 1*10^7^ CFU/mouse at 7 d.p.i.

f Group infected with *S.* Enteritidis 327 lux and bacteriophage treated with a single dose of 1*10^14^ CFU/mouse at 7 d.p.i.

g Group infected with *S.* Enteritidis 327 lux and bacteriophage treated with a dose of 1*10^7^ PFU/mouse each day starting at 7 d.p.i.

h Group infected with *S.* Enteritidis 327 lux and bacteriophage treated with a dose of 1*10^14^ PFU/mouse each day starting at 7 d.p.i.

i Group infected with *S.* Enteritidis 327 lux and bacteriophage treated with a dose of 1*10^7^ PFU/mouse 1 h after bacteria administration and subsequently each day starting at 7 d.p.i.

j Group infected with *S.* Enteritidis 327 lux and bacteriophage treated with a dose of 1*10^14^ PFU/mouse 1 h after bacteria administration and subsequently each day starting at 7 d.p.i.

k Group infected with *S.* Enteritidis 327 lux and bacteriophage treated with a dose of 1*10^7^ PFU/mouse 1 h after bacteria administration and each day post-infection.

l Group infected with *S.* Enteritidis 327 lux and bacteriophage treated with a dose of 1*10^14^ PFU/mouse 1 h after bacteria administration and each day post-infection.

m Untreated control.

n d.p.i., days post-infection.

Animals were monitored every morning for bioluminescent signals using the NightOwl 983 imaging system (Berthold, Bad Wildbad, Germany) as described elsewhere (Kuźmińska-Bajor et al., 2015). Briefly, to confirm that the *in vivo* bioluminescence signals and changes in physical appearance accurately represent the presence of the bacterial load, as the bioluminescent signals, accompanied by clinical symptoms were observed, the mice were sacrificed and their livers, spleens and lungs were analyzed for the presence of CFU. The organs were homogenized in PBS, properly diluted, and plated on LB agar with 0.2 mg/ml erythromycin for bacterial number determination. The bioluminescent signals were always accompanied by the presence of *S.* Enteritidis 327 lux strain CFU in analyzed tissue samples, and CFU were not observed in livers, spleens and lungs isolated from mice that did not develop such signals.

### Bacteriophage therapy trials in the experimental chicken model

For the *in vivo* experiments on experimentally infected chickens, 7-day-old, *Salmonella*-free ROSS 308 broilers obtained from a local farm were divided into four groups (10 birds/group) and housed separately in wire cages at an ambient temperature of 30°C. Sterile chicken feed Brojler Grower II (Tasomix, Poland) and water were provided *ad libitum*. All experimental work involving birds was approved by the Local Ethics Committee for Animal Experimentation (protocol code 114/2015; Wrocław, Poland). Group 1 was a positive control for *Salmonella* infection and birds were challenged directly to the crop with 10^5^ CFU of *S.* Enteritidis 327 lux strain in 1 ml of PBS per bird. Group 2 received only the UPWr_S134 phage cocktail at 1, 4, 7, 10 and 13 days of the experiment. Group 3 was infected with 10^5^ CFU/ml of *S.* Enteritidis 327 lux strain per bird and inoculated with a pool of 1 × 10^7^ PFU of each phage from the UPWr_S134 phage cocktail. At 1, 4, 7, 10 and 13 days post-infection (d.p.i.) birds from group 3 were treated with the UPWr_S134 phage cocktail. Group 4 was kept as an uninfected control (Table 2). On the 14th d.p.i., all birds were necropsied and attempts were made to isolate *S.* Enteritidis 327 lux strain and bacteriophages from the internal tissues of every bird. The spleen, liver, bursa of Fabricius and cecal tonsils were removed aseptically and the colonization level was assessed by estimating the number of CFU and PFU. The cecal tonsils and cecal contents were weighed, and homogenized in 0.2 ml of PBS. The bursa of Fabricius, spleen and liver were weighed after washing with PBS, and each organ was homogenized with 5, 5, and 50 ml of cold PBS, respectively. Ten-fold dilutions of homogenates were plated onto LB agar containing erythromycin (0.2 mg/ml) and plates were incubated overnight at 37°C. Identification of *Salmonella* was carried out by bioluminescence imaging and luminescent colonies were counted using bioluminescence imaging. For the PFU estimation tissue homogenates were centrifuged at 4000 × *g* to remove solid particles and then supernatants were filtered through 0.22 μm filters. The phage titration was estimated using a double agar layer plaque assay method. The mean CFU and PFU per 1 g of each tissue were calculated.

**Table 2:** Chicken experimental design.

Group^a^	Doses		Treatment schedule of bacteriophages [d.p.i.]^f^
	UPWr_S134	*Salmonella* Enteritidis 327 lux	
1^b^	–	1*10^5^ CFU/ml	–
2^c^	3*10^10^ PFU/ml	–	1,4,7,10,13
3^d^	3*10^10^ PFU/ml	1*10^5^ CFU/ml	1,4,7,10,13
4^e^	–	–	–

UPWr_S134 phage cocktail and *Salmonella* Enteritidis 327 lux were administered directly to the crop.

a Each group consisted of 10 chickens.

b 1 control positive, infected with *Salmonella* Enteritidis 327 lux.

c 2 infected with *S.* Enteritidis 327 lux and bacteriophage treated.

d 3 control negative, bacteriophage treated.

e 4 untreated control.

f d.p.i., days post-infection.

### Statistical analysis

All statistical tests were performed on log_10_ transformed data using STATISTICA version 13 software (TIBCO Software Inc.). To examine the variance in the titer of UPWr_S134 phage cocktail incubated in different pH and different temperature points and between treatments, the analysis of variance (ANOVA) followed by Tukey’s *post-hoc* test was applied. Student’s *t*-test was employed for statistical analysis of data obtained from *in vitro* assay in simulated gastric juice. To confirm that the UPWr_S134 phage cocktail treatment is a factor decreasing the colonization potential of *S.* Enteritidis, we compared the infection-free times of mice inoculated with *S.* Enteritidis 327 lux with or without phage treatment. Infection-free curves were obtained using the method of Kaplan and Meier, and the significance of differences was determined by the Gehan–Breslow–Wilcoxon test. Colonization of internal organs in both mouse and chicken models was analyzed using the Mann–Whitney *U* test with the non-Gaussian distribution. A *value of p* of < 0.05 was considered to be significant. All data were analyzed using two-tailed tests. To compare bacterial load (CFU) and phage load (PFU) recovered from chickens’ internal organs the nonparametric Mann–Whitney U-test was employed.

## Results

### UPWr_S134 phage stability at different temperatures

**Figure 1:**
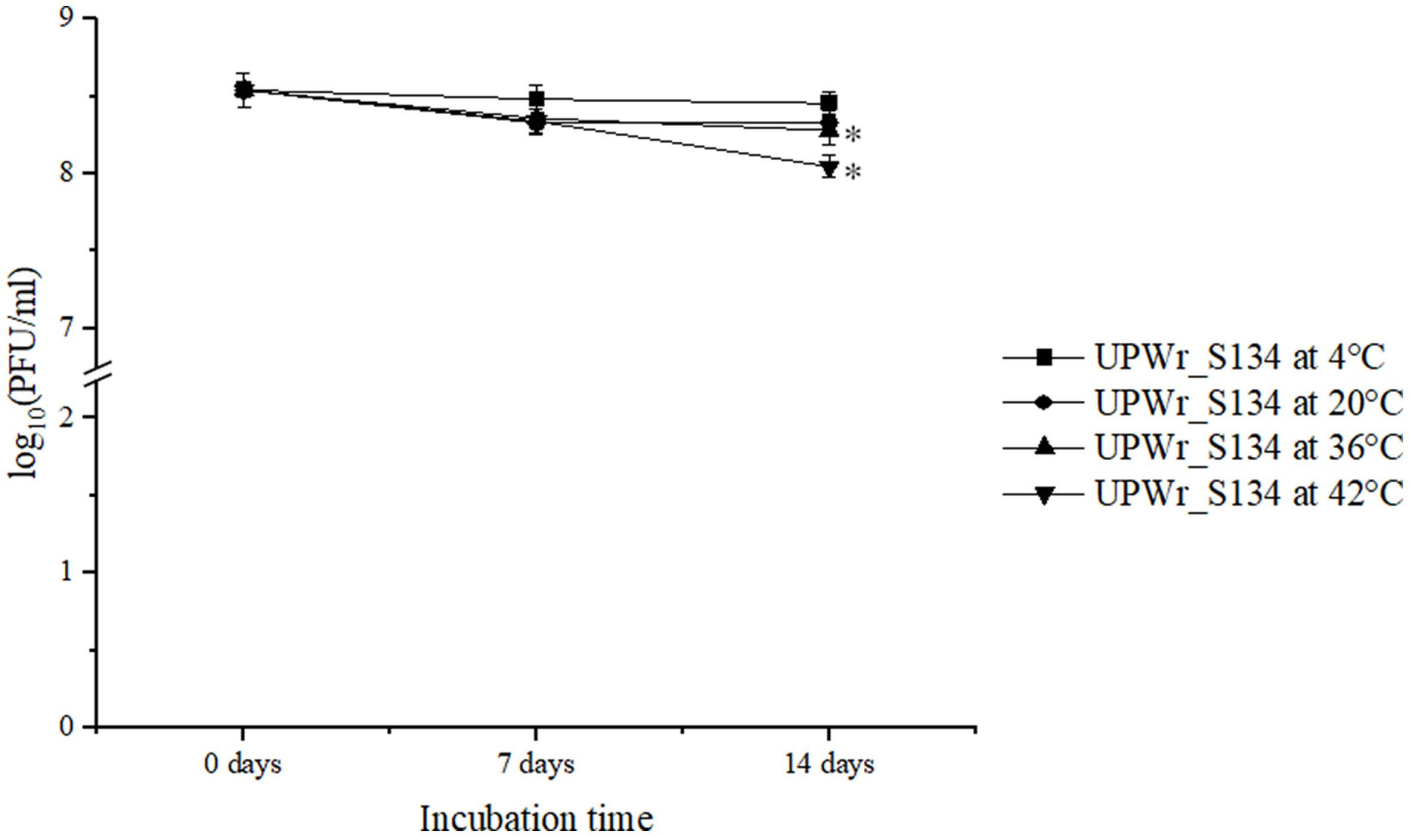
Effect of temperature on the stability of UPWr_S134 phage cocktail. UPWr_S134 phage cocktail was incubated for 2 weeks at four different temperatures. Each data point represents the mean of three independent experiments. ^*^ represents *p* < 0.01 and indicates a significant difference between experimental groups.

The UPWr_S134 phage cocktail was tested for 2-week stability at four temperatures, 4, 20, 36 and 42°C, and found to be stable at all tested conditions (Figure 1). Storage at 4°C had a negligible effect on the titer of phages (*p* < 0.05). The influence of 20°C within 2 weeks, and 36 and 42°C within 1 week on the titer of the phage cocktail was also negligible, but the phage titer showed a slight decrease after 2 weeks of incubation at temperatures of 36 and 42°C estimated to be 0.18 (*p* < 0.05) and 0.41 PFU/ml (*p* < 0.05), respectively.

### UPWr_S134 phage cocktail stability at acidic and alkaline pH

**Figure 2:**
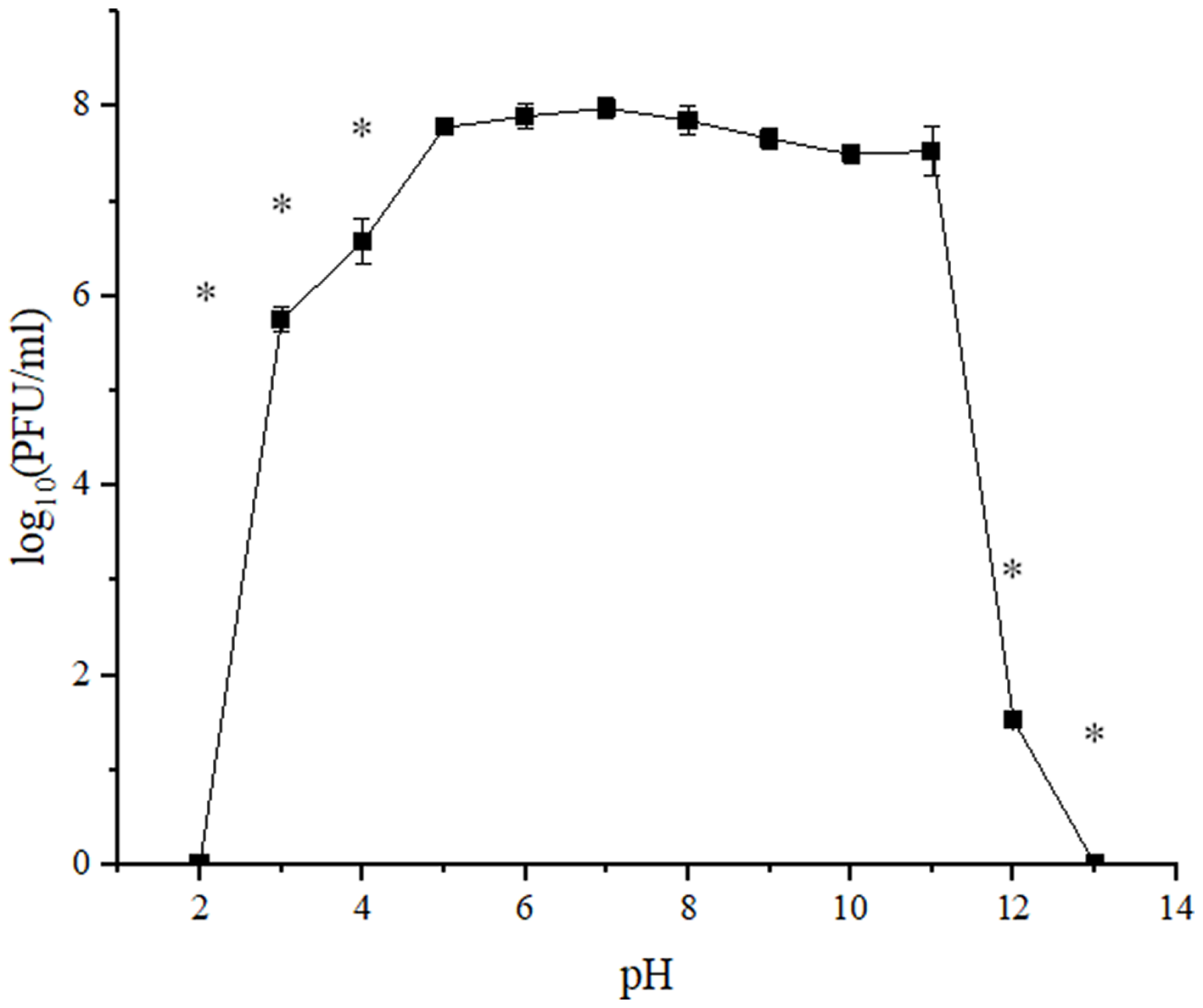
pH stability of UPWr_S134 phage cocktail. Phage infectibility was estimated at a pH ranging from 2 to 13 after 1 h of incubation. Values represent the mean with a standard deviation of three replicates. ^*^ represents *p* < 0.01 and indicates a significant difference between experimental groups.

The UPWr_S134 phage cocktail was tested against a wide pH range (2–13) during 1-h incubation, to determine its stability under acidic and alkaline conditions (Figure 2). In the acidic pH range (2–5), phages exhibited the most stability at pH 5, with minimal loss in viability of only 0.2 logs PFU/ml after 1 h of incubation (*p* > 0.01). Phages included in the UPWr_S134 cocktail survived at pH 3 and 4 for 1 h maintaining high activity estimated to be 5.7 and 6.6 log_10_ PFU/ml, respectively, which corresponds to 72 and 82.4%, respectively, compared to the control. However, they did not survive at pH 2 over 1 h (Figure 2). Phages survived well at pH 7, 8, and 9 with no significant loss in viability after incubation (*p* > 0.01). Phages included in the UPWr_S134 cocktail were stable at pH 9, 10, and 11 with slight reductions in the population of about 4, 6, and 6%, respectively, during the incubation (*p* > 0.01), respectively. A significant phage titer reduction was observed for pH 12 and the number of phages was calculated to be about 19% of the control phage cocktail (*p* < 0.01).

### Assessment of UPWR_S134 phage cocktail survival in the SGF model

**Figure 3:**
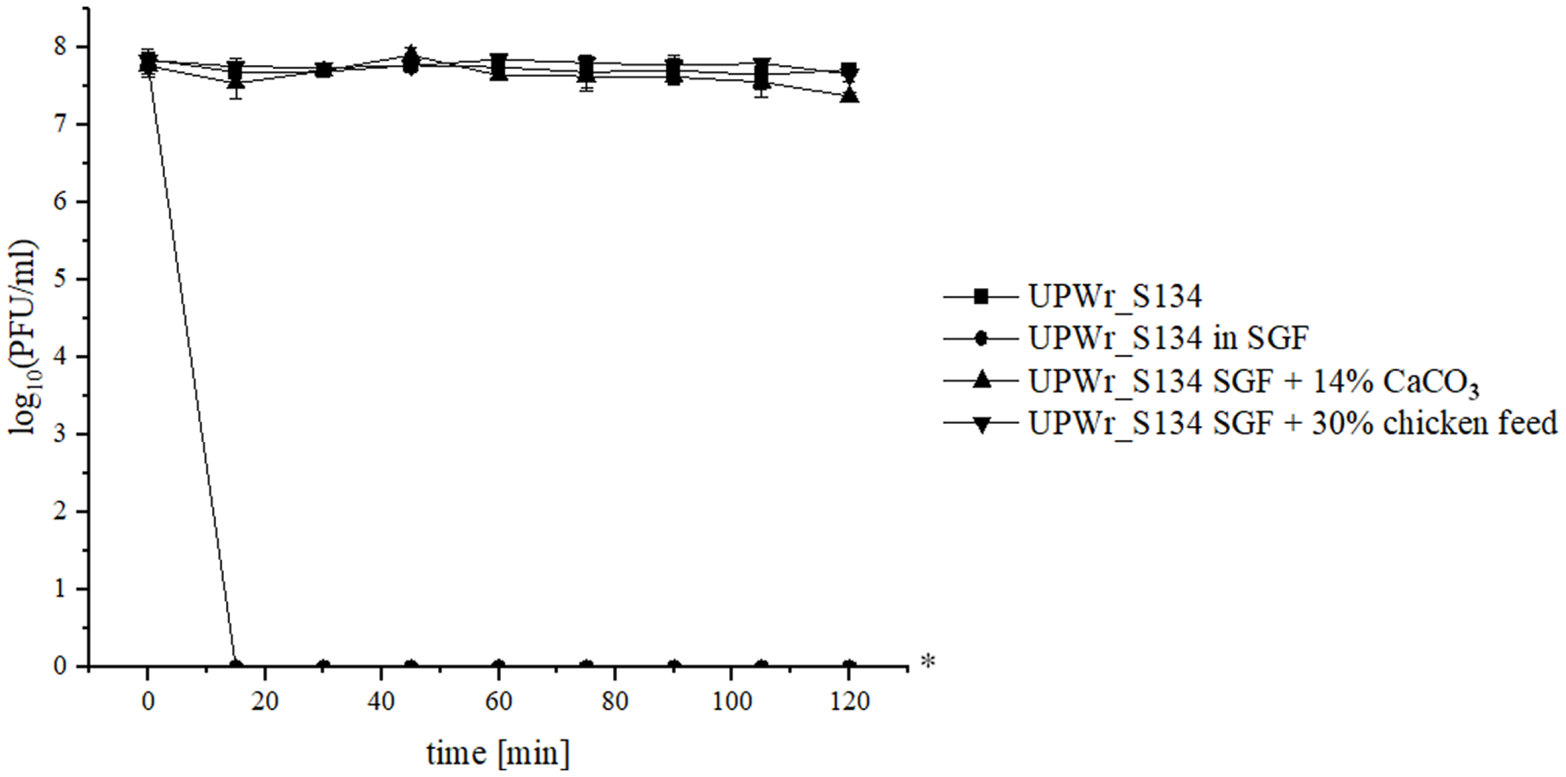
UPWr_S134 phage cocktail activity under simulated gastric conditions. Effect on UPWr_S134 phage cocktail activity in simulated gastric fluids (SGF; squares), SGF with 14% CaCO_3_ (triangles), and SGF with the addition of 30% chicken feed. ^*^ represents *p* < 0.01 and indicates a significant difference between experimental groups.

UPWr_S134 phage cocktail’s ability to endure intragastric conditions was tested *in vitro*. The phages were very sensitive to low pH and the pepsin activity of control SGF and completely inactivated following 15 min of incubation at 42°C corresponding to the chicken deep body temperature (Figure 3). However, the addition of the antacid 14% CaCO_3_ resulted in the complete neutralization of the negative influence of SGF conditions on phages (*p* < 0.01). We also performed the experiment of suppressing stomach acidity and pepsin activity in relation to phage survival by adding 30% chicken feed and we found that the adverse effect of SGF on the UPWr_S134 phage cocktail was completely abolished (*p* < 0.01; Figure 3).

### UPWr_S134 efficacy in the mouse model

**Figure 4:**
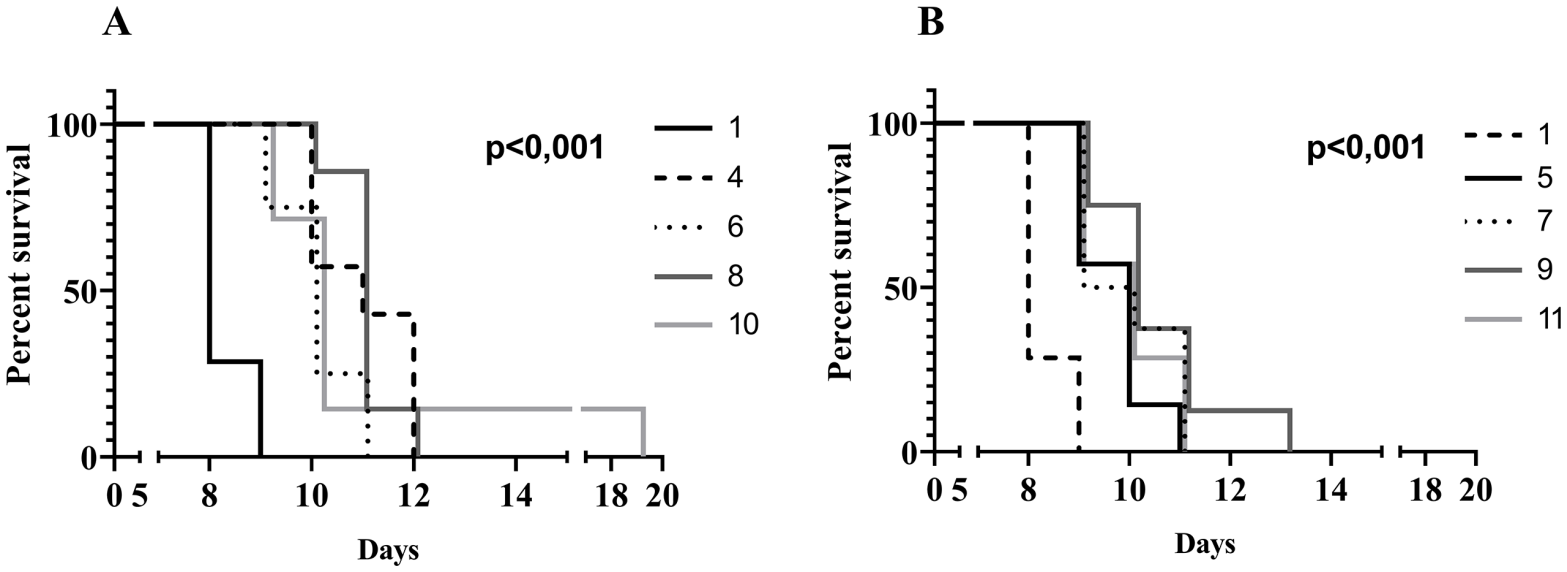
Kaplan–Meier survival analysis illustrating infection of mice with *Salmonella* Enteritidis 327 lux and treated with UPWr_S134 phage cocktail. Survival curves of *Salmonella*-infected mice (black solid line, group 1) and *Salmonella*-infected mice treated with UPWr_S134 phage cocktail at dose of 1*10^7^ PFU/mouse administered according to different schedules: at 7 days post-infection (d.p.i.) with a single dose (black dashed line, group 4); each day starting at 7 d.p.i. (light grey dotted line, group 6); 1 h after infection and subsequently each day starting at 7 d.p.i. (dark grey solid line, group 8) and 1 h after infection and each day post-infection (light grey solid line, group 10) **(A)**. Survival curves of *Salmonella*-infected mice (black dashed line, group 1) and *Salmonella*-infected mice treated with UPWr_S134 phage cocktail at dose of 1*10^14^ PFU/mouse administered according to different schedules: at 7 d.p.i. with a single dose (black solid line, group 5); each day starting at 7 d.p.i. (light grey dotted line, group 7); 1 h after infection and subsequently each day starting at 7 d.p.i. (dark grey solid line, group 9) and 1 h after infection and each day post-infection (light grey solid line, group 11) **(B)**. The survival curves were plotted using the Kaplan–Meier method, and the log-rank test was used to analyze the difference in survival rates in GraphPad Prism 7.0. *p* < 0.001 and indicates a significant difference between groups infected with *S.* Enteritidis and treated with the UPWr_S134 phage cocktail.

**Figure 5:**
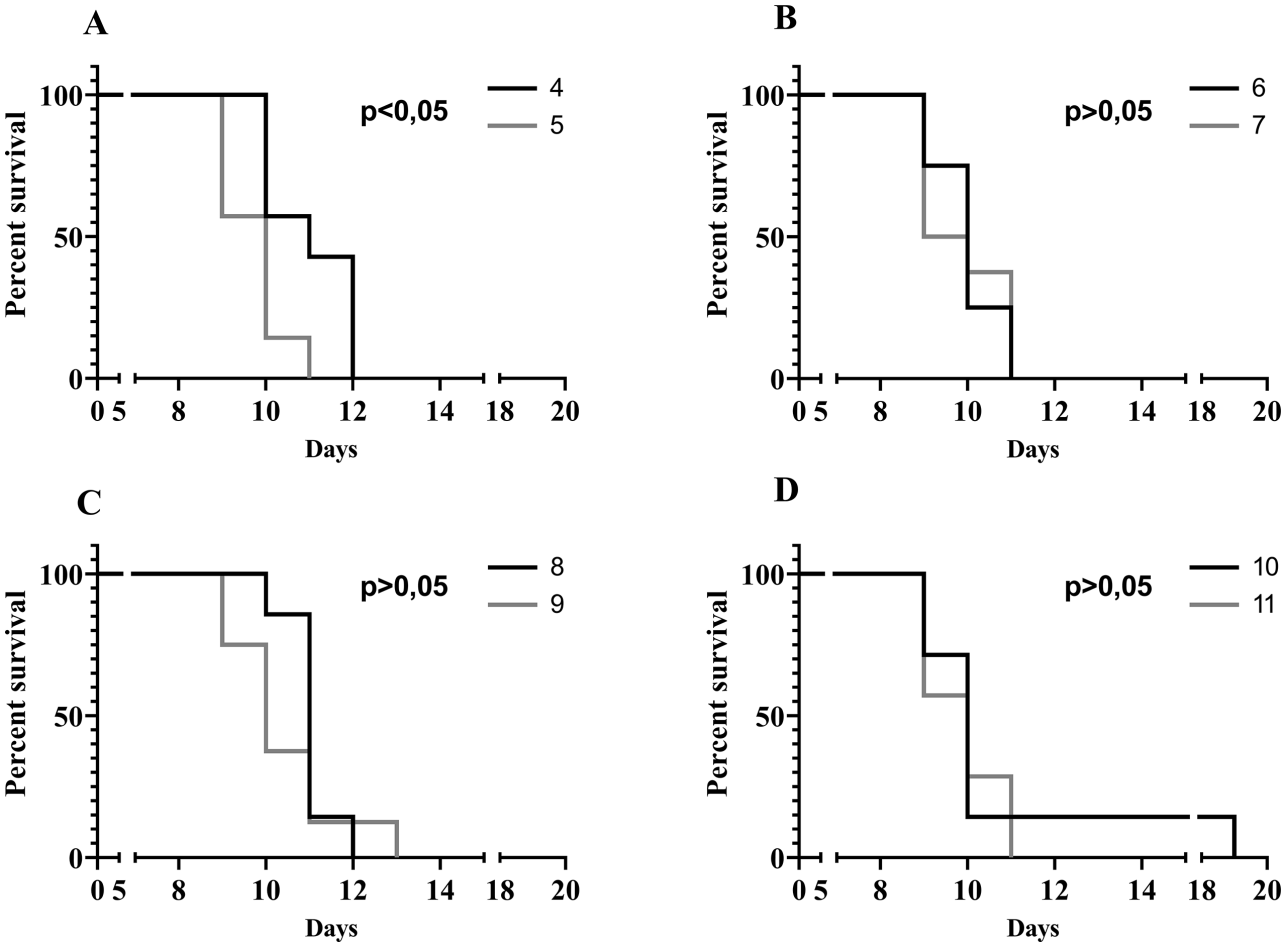
Kaplan–Meier survival analysis illustrating infection of mice with *Salmonella* Enteritidis 327 lux and treated with UPWr_S134 phage cocktail. Survival curves of *Salmonella*-infected mice treated with UPWr_S134 phage cocktail administered at 7 d.p.i. by intragastric inoculation with a single dose of 1*10^7^ PFU/mouse (black line) and 1*10^14^ PFU/mouse (grey line) **(A)**. Survival curves of *Salmonella*-infected mice treated with UPWr_S134 phage cocktail administered each day starting at 7 d.p.i. by the intragastric inoculation with a dose of 1*10^7^ PFU/mouse (black line) and 1*10^14^ PFU/mouse (grey line) **(B)**. Survival curves of *Salmonella*-infected mice treated with UPWr_S134 phage cocktail administered 1 h after infection and subsequently each day starting at 7 d.p.i. by intragastric inoculation with a dose of 1*10^7^ PFU/mouse (black line) and 1*10^14^ PFU/mouse (grey line) **(C)**. Survival curves of *Salmonella*-infected mice treated with UPWr_S134 phage cocktail administered 1 h after infection and each day post-infection by intragastric inoculation with a dose of 1*10^7^ PFU/mouse (black line) and 1*10^14^ PFU/mouse (grey line) **(D)**. The survival curves were plotted using the Kaplan–Meier method, and the log-rank test was used to analyze the difference in survival rates in GraphPad Prism 7.0.

To evaluate the *in vivo* effectiveness of the UPWr_S134 phage cocktail, a mouse model of acute typhoid fever caused by the *S.* Enteritidis 327 lux strain was applied. According to all the results presented in Table 3 and Figure 4, we found significant differences in colonization patterns between *Salmonella*-infected mice and mice from all groups treated with the UPWr_S134 phage cocktail. Oral administration of a dose of 10^7^ of *S.* Enteritidis CFU caused development of clinical symptoms in animals from control group 1, indicating murine typhoid like-disease by days 8 and 9. Mice administered with a single dose of 10^7^ or 10^14^ PFU of the phage cocktail UPWr_S134 on the 7th day post-infection with *S.* Enteritidis (groups 4 and 5, respectively) survived significantly longer than mice in the control group (*p* < 0.001). In group 4, 3 mice died on day 10, 1 mouse on day 11 and 3 mice on day 12, in group 5, 3 mice died on day 9, another 3 at 10 and 1 on day 11. A similar effect was seen in mice treated daily starting from the 7th day after *S.* Enteritidis 327 lux infection with both of the phage cocktail doses and with a close treatment schedule additionally treated 1 h post-infection (groups 8 and 9). In group 8, 1 mouse, 5 mice and 1 mouse died on days 10, 11 and 12, respectively. Whereas in group 9, on days 9, 10, 11 and 13 died 2, 3, 2 mice and 1 mouse, respectively. The difference in mice lifespan between the phage-treated groups 8 and 9 and control group 1 was also significant with *p* < 0.001. For groups 10 and 11 in which mice received phages daily starting on the 1st day post-infection at doses 10^7^ and 10^14^ PFU/mouse, respectively, the levels of statistical significance were set as *p* < 0.001 and *p* < 0.05, respectively. In the comparison of groups receiving different doses of UPWr_S134 phage cocktail according to the same treatment program, a significant difference was found only for the single-dose treatment schedule of groups 4 and 5 treated with 10^7^ and 10^14^ PFU per animal with *p* > 0.001, indicating greater effectiveness of the lower dose of the UPWr_S134 cocktail (Figure 5A). For the remaining 3 types of treatment schedules, no significant differences were found between 10^7^ and 10^14^ PFU/mouse doses (Figures 5B–D).

**Table 3:** Deaths that occurred in groups of mice infected with *Salmonella* Enteritidis 327 lux and given different treatments of UPWr_S134 phage cocktail at different times after challenge with *Salmonella*.

Group no.	Day of euthanasia
1	8, 8, 8, 8, 8, 9, 9
2	21, 21, 21, 21, 21, 21, 21
3	21, 21, 21, 21, 21, 21, 21
4^***^	10, 10, 10, 11, 12, 12, 12
5^***^	9, 9, 9, 10, 10, 10, 11
6^***^	9, 9, 10, 10, 10, 11, 11
7^***^	9, 9, 9, 9, 10, 11, 11, 11
8^***^	10, 11, 11, 11, 11, 11, 12
9^***^	9, 9, 10, 10, 11, 11, 13
10^***^	9, 9, 10, 10, 10, 10, 19
11^*^	9, 9, 9, 10, 10, 11, 11
12	21, 21, 21, 21, 21, 21, 21

^***^ represents *p* < 0.001, ^*^ represents *p* < 0.05 and indicates a significant difference between experimental groups and group 1.

All of the challenged mice were sacrificed between bioluminescence signal detection and developing typhoid-like symptoms and the concentrations of strain *S.* Enteritidis 327 lux were determined in their livers, spleens, and lungs. The CFU of the *Salmonella* strain showed no statistically significant difference and were estimated to be 8.9 ± 2.82 log_10_CFU in lungs, 10.1 ± 1.67 log_10_CFU in spleens and 8.1 ± 2.11 log_10_CFU in livers (*p* > 0.05).

### Chicken model

**Figure 6:**
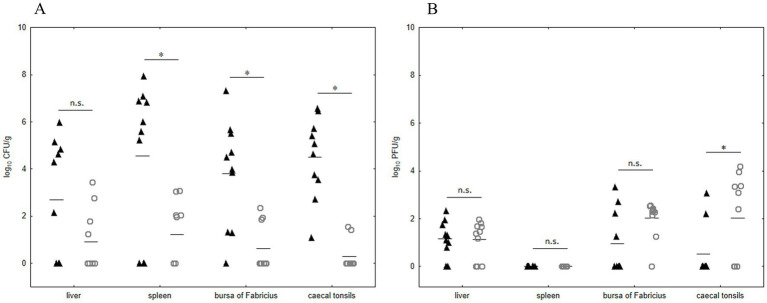
Effect of phage cocktail UPWr_S134 on *Salmonella* Enteritidis 327 lux number in internal organs in an experimental chicken model. Results for bacterial load are shown as counts for individual animals plus the median (*n* = 10 per group) of control group 1 (black triangle) infected with *S.* Enteritidis 327 lux and group 3 (empty circle) infected with *S.* Enteritidis 327 lux treated with phage cocktail UPWr_S134 **(A)**. Results for phage load are shown as counts for individual animals plus the median (*n* = 10 per group) for control group 2 (black triangle) treated with phage cocktail UPWr_S134 and the group 3 (empty circle) infected with *S.* Enteritidis 327 lux treated with phage cocktail UPWr_S134 **(B)**. ^*^ represents *p* < 0.05 and indicates a significant difference between groups, n.s., not significant.

To evaluate the effectiveness of the UPWr_S134 phage cocktail in poultry we used chickens experimentally infected with *Salmonella*. We examined the *S.* Enteritidis 327 lux load in the liver and lymphoid organs such as the spleen, the bursa of Fabricius, and the cecal tonsils at 14 d.p.i. Significant differences in *Salmonella* number between chickens infected with *S.* Enteritidis 327 lux from phage-treated group 3 and untreated birds (group 1) were observed in all lymphoid organs *p* < 0.05 (Figure 6A). No significant difference between group 1 and phage-treated group 3 was recorded for birds colonized in liver samples taken during necropsy (*p* > 0.05).

In comparison to group 2, the number of phages isolated from group 3 was significantly higher only in cecal tonsils (*p* < 0.05). In the bursa of Fabricius and liver samples of birds from *Salmonella*-infected group 3, in comparison to group 2, there was a higher number of organs positive for phage presence, with median values of 2.31 and 1.42, respectively. These differences were not statistically significant (*p* > 0.05; Figure 6B). Likewise, for liver samples median values of 1.21 and 1.42, for group 2 and 3, respectively were statistically insignificant (*p* > 0.05). No bacteriophages were detected in spleen samples taken from birds among both groups treated with phages (groups 2 and 3). Internal organ samples of control birds from group 4 were negative for both bacteriophages and *Salmonella*.

## Discussion

Constantly growing consumption of poultry products resulted in intensive farming. It leads, on the one hand, to the frequent occurrence of highly important zoonotic bacterial pathogens such as *Salmonella*, and on the other hand, to overuse and misuse of antimicrobials. Animal production practices are associated with the use of antibacterial drugs used in human therapy including colistin, considered critically important for treating life-threatening infections and called a “last hope” antibiotic. Emerging antimicrobial resistance of *Salmonella* isolated from poultry, is exacerbating the problem of infection with these pathogen (de Mesquita Souza Saraiva et al., 2022). Thus, many efforts are taken to eliminate *Salmonella* from the food chain. Constant attempts to solve these problems are made using approaches with ecological and environmental significance, and phages are one of the most promising tools to control pathogenic and drug-resistant bacteria in poultry production. According to several recent reviews, there is much evidence indicating the effectiveness of phages in the great reduction of *Salmonella* in poultry (Loponte et al., 2021; Khan and Rahman, 2022; Ruvalcaba-Gómez et al., 2022). Recently, new phage-based commercial products reviewed elsewhere (Żbikowska et al., 2020) have been registered and their commercial applications in poultry housing are being explored.

However, to state unequivocally that phages are safe and could be ordinarily applied in poultry-intensive production it is necessary to collect data from many multifaceted studies to assess the hallmark of the *Salmonella*-targeting phages’ activity. Previously, we isolated and characterized five phages infecting different *Salmonella* serovars (Kuźmińska-Bajor et al., 2021) and we confirmed the UPWr_S134 phage cocktail’s ability to eliminate *S.* Enteritidis from abundant multispecies biofilm formed on the poultry drinkers’ surface in an *in vivo* chicken model (Korzeniowski et al., 2022). In order to test the suitability of the phage cocktail for use on the farm, it is important to define the method of administration to the chickens taking into account resistance to adverse environmental conditions and financial costs. Phages in the cocktail UPWr_S134 showed the ability to remain active after storage at various temperatures which reflect different conditions on farms and in live animals. UPWr_S134 retained high activity with a wide pH range, allowing for on-farm use as a water additive, which usually contained acidifiers and pH close to 4.5 to eliminate pathogenic bacteria from the water. In such conditions, the number of UPWr_S134 phage cocktail was high enough to effectively combat *Salmonella*. Moreover, phages for practical applications must withstand the proteolytic environment and low pH of the stomach juice, which is typically in the range of 2.1–3.6 (Colom et al., 2017). The use of antacids such as calcium carbonate or bicarbonate in order to neutralize stomach acidity has been common practice in several studies (Tanji et al., 2005; Atterbury et al., 2007; Nale et al., 2016; Colom et al., 2017; Yen et al., 2017). In fact, phages included in the UPWr_S134 cocktail completely lose their ability to infect *Salmonella* after incubation in simulated gastric conditions, confirming the previous reports where phages were recovered after exposure to SGF (Mhone et al., 2022). However, the addition of calcium carbonate resulted in a protective effect on phage activity, confirming previous studies (Koo et al., 2000; Ma et al., 2008). From the practical and economic point of view, at the farm level phages should be administered with drinking water and potential costs of other additives should be reduced. Therefore, we examined the survivability of phages in the cocktail UPWr_S134 in SGF with the addition of chicken feed. This approach is likely a reflection of chicken stomach contents. Our results indicated that the presence of feed in gastric juice allows the maintenance of the UPWr_S134 phage cocktail’s activity when administered to chicken *via* the oral route.

Although mice do not normally get diarrhea as a result of infection with *S.* Enteritidis and develop a severe systemic disease (Kuźmińska-Bajor et al., 2015), the murine infection model is commonly applied in research on the ability of phages to combat *Salmonella* (reviewed in Penziner et al., 2021). In an acute infection model, the UPWr_S134 phage cocktail delayed symptoms of intrinsic infection in all analyzed treatment schedules. These findings revealed high anti-*Salmonella* activity of the UPWr_S134 phage cocktail at a titer of 10^7^ PFU in live animals. In studies on a murine model, phages had a different effect on *Salmonella* clearance causing complete pathogen elimination (Capparelli et al., 2010; Liu et al., 2022), or delaying the burden of bacteria in mice after intraperitoneal administration (Lamy-Besnier et al., 2021), or had no influence on the number of *Salmonella* and course of infection (Bardina et al., 2012). Importantly, it was noted that the effect of the very high dose of 10^14^ PFU of UPWr_S134 phage cocktail in delaying sepsis was comparable to the dose of 10^7^ PFU except for single phage treatment at 7 d.p.i. We speculate that this situation can be explained by the phenomenon of “lysis from without” when an overload of phages at high titer infects bacterial cells unspecifically and causes cell wall destruction (Matsuzaki et al., 2003; Abedon, 2011). Similar effect was revealed for daily treatment schedule starting on the 1 day p.i., where the difference was still significant, albeit at a slightly lower significance level. Comparable effect of both phage cocktail doses 10^7^ and 10^14^ PFU indicated that an increase in UPWr_S134 phage cocktail titer is unnecessary. This outcome is significant in light of the prospective design and UPWr_S134 phage cocktail dosage for practical application against *Salmonella*. However, UPWr_S134 phage cocktail is potentially intended for use in poultry, hence experimentally infected chickens are a more appropriate model. In the chicken infection model, we observed that the UPWr_S134 phage cocktail effectively decreased the number of *Salmonella*-positive organs and pathogen load of internal organs such as spleen, liver, bursa of Fabricius and cecal tonsils. Similar results have been observed by other authors. After 6 d.p.i. the number of *Salmonella*-infected internal organs such as spleen, liver, heart and cecum was significantly lower than in chickens that did not receive phages like the number of *S*. Typhimurium in cecal content (Wong et al., 2014). Likewise, Bardina et al. (2012) reported that phage treatment of chickens experimentally infected with *Salmonella* Typhimurium reduced by 2 log_10_ the number of pathogens in the cecum in comparison to untreated birds. Related conclusions were reached by Nabil et al. (2018) for cecal contents of chickens infected with *S.* Enteritidis and *S.* Typhimurium. In this study, both the number of *Salmonella* and the number of positive birds was significantly lower. Our results from *in vivo* studies on both chicken and murine models demonstrate that the UPWr_S134 phage cocktail could be an effective tool against Salmonella in the poultry industry. However, further research is needed to assess the relationship between the use of phages and the productivity parameters such as feed conversion, weight gain, and weight homogeneity to expand the current knowledge about the performance of the UPWr_S134 phage cocktail under commercial poultry housing.

## Conclusion

This study revealed that the UPWr_S134 phage cocktail is an effective tool against *S.* Enteritidis. The polyvalent UPWr_S134 phage cocktail can maintain activity after storage under temperatures representing temperatures of storage conditions, broiler handling, and the chicken body and exhibited robust pH stability. Although the activity of the UPWr_S134 phage cocktail in SGF was completely lost, the presence of chicken feed enabled the maintenance of the ability to combat *Salmonella*, confirming the great effectiveness of this cocktail. High anti-*Salmonella* effectiveness was also corroborated in experimentally infected mice and broiler chickens. Thus, these observations place the UPWr_S134 phage cocktail among effective biocontrol agents in combating *S.* Enteritidis in broiler chickens.

## Data availability statement

The raw data supporting the conclusions of this article will be made available by the authors, without undue reservation.

## Ethics statement

The animal study was reviewed and approved by Bioethics Committee of the Hirszfeld Institute of Immunology and Experimental Therapy. Written informed consent was obtained from the owners for the participation of their animals in this study.

## Author contributions

MK-B conceived and supervised the studies and made substantial inputs into the analysis and all drafts, obtained funding, and was a major contributor to writing the manuscript. PŚ performed the statistical analysis, drafted the figures and co-drafted the manuscript. PK performed the analysis of phages in different pH and in the SGF model, provided assistance throughout the study on the chicken model and conducted microbiological analysis of dead chicken. MK performed and supervised *in vivo* trials in the chicken model. DM provided assistance throughout the study on mouse model and conduct microbiological analysis of dead mice. AW-B performed mice necropsy and material sampling. EŚ performed the analysis of phages stability at different temperatures. KG performed and supervised the study on the mouse model performed the statistical analysis and drafted the figures. All authors contributed to the article and approved the submitted version.

## Funding

This study was supported by the National Centre for Research and Development, LIDER program no. LIDER/378/L-6/14/NCBR/2015. The APC was co-financed by Wroclaw University of Environmental and Life Sciences and University of Wroclaw and Biotechnologists Student Science Club.

## Conflict of interest

The authors declare that the research was conducted in the absence of any commercial or financial relationships that could be construed as a potential conflict of interest.

## Publisher’s note

All claims expressed in this article are solely those of the authors and do not necessarily represent those of their affiliated organizations, or those of the publisher, the editors and the reviewers. Any product that may be evaluated in this article, or claim that may be made by its manufacturer, is not guaranteed or endorsed by the publisher.
